# Life's Essential 8 score and arterial stiffness in patients with type 2 diabetes

**DOI:** 10.20945/2359-4292-2026-0117

**Published:** 2026-10-11

**Authors:** Kangbo Li, Xinyu Wu, Qi Qi, Philipp Hillmeister, Ivo Buschmann, Shouling Wu, Quanle Han

**Affiliations:** 1 School of Clinical Medicine, North China University of Science and Technology, Tangshan, China; 2 Department of Cardiology, Tangshan Gongren Hospital, Tangshan, China; 3 School of Clinical Medicine, Hebei Medical University, Shijiazhuang, China; 4 Department for Angiology, Center for Internal Medicine I, University Clinic Brandenburg, Brandenburg Medical School Theodor Fontane, Brandenburg an der Havel, Germany; 5 Department of Cardiology, Kailuan General Hospital, Tangshan, China

**Keywords:** Cardiovascular health, arterial stiffness, brachial-ankle pulse wave velocity, type 2 diabetes

## Abstract

**Objective:**

Research examining the connection between Life's Essential 8 score (LE8) and arterial stiffness (AS) in patients with diabetes is currently insufficient. This study aimed to investigate the relationship between cardiovascular health and the development of AS in patients with type 2 diabetes (T2D) in a cohort study.

**Subjects and methods:**

A total of 2,685 patients with T2D were enrolled in this study. All patients were divided into four groups based on quartiles of cumulative exposure of LE8 (cum-LE8) score. The study endpoint was increased AS (iAS), which was defined as a brachial – ankle pulse wave velocity ≥ 1800 cm/s. The Kaplan-Meier method was used to determine the cumulative incidence of iAS, while the log-rank test was utilized to evaluate the differences among groups. Cox proportional hazards regression model was applied to investigate the impact of various cum-LE8 groups on the risk of iAS.

**Results:**

Over a median follow-up of 9.76 years, we identified 1,375 cases of iAS. The incidence rate of iAS in the quartile 1-4 were 71.02 (64.24-78.52), 67.68 (61.13-74.94), 56.05 (50.28-62.47) and 50.47 (45.05-56.53) per 1000 person-years, respectively. The cumulative incidence of iAS was highest in quartile 1 and gradually decreased from quartile 1 to quartile 4 (log-rank *P* < 0.01). Compared with quartile 1, the hazard ratios (95% confidence intervals) of quartile 2-4 were 0.69 (0.59-0.80), 0.41 (0.35-0.49), and 0.15 (0.12-0.18), respectively.

**Conclusion:**

Attaining a superior long-term cardiovascular health status is related to a reduced risk of iAS.

## INTRODUCTION

The global incidence of diabetes mellitus is significantly increasing. Projections indicate that the number of individuals affected by this condition may reach 643 million (11.3%) by 2030 (^1^). Individuals diagnosed with diabetes are at increased risk of vascular diseases, with cardiovascular diseases (CVDs) being the leading cause of mortality in this population (^2^). However, the underlying pathophysiological mechanisms linking these conditions remain only partially understood. One potential pathway connecting diabetes to elevated cardiovascular risk is arterial stiffness (AS) (^3^), which is characterized by reduced arterial compliance, diffuse dilation, and hypertrophy of large arteries, resulting in a loss of elasticity (^4^). Variations in AS occur across different regions and types of arteries, and recent research has identified it as a prognostic marker for coronary artery disease and peripheral artery disease (^5^,^6^).

AS is a prominent characteristic of vascular complications associated with diabetes (^7^). Emerging evidence suggests that even individuals in pre-diabetic states, such as insulin resistance (IR), exhibit signs of abnormal AS (^8^,^9^,^10^). IR contributes to endothelial dysfunction by disrupting nitric oxide (NO)-mediated vasodilation (^11^). Under IR condition, activation of NO synthase is reduced, while superoxide production is elevated. The interplay of these factors ultimately decreases NO bioavailability (^12^). Notably, individuals with diabetes and microvascular disease show lower basal NO levels than those without microvascular disease, with progressively greater reductions observed as disease severity increases (^13^).

A healthy lifestyle is widely acknowledged as an effective strategy to reduce the incidence of and mortality from CVDs (^14^). In 2010, the American Heart Association (AHA) launched "Life's Simple 7" (LS7) to promote cardiovascular health (CVH). This initiative emphasizes the management of cholesterol, blood pressure, glucose levels, smoking habits, physical activity, dietary choices, and body mass index (BMI) (^15^). In 2022, AHA introduced a new framework for defining and quantifying LS7 metrics, termed "Life's Essential 8" (LE8) (^16^). Recent studies have investigated the potential relationship between LE8 and CVD risk, suggesting that LE8 may serve as a comprehensive metric and guideline for identifying populations at elevated risk during the preclinical stage of CVDs (^17^). However, research investigating the association between LE8 and increased AS (iAS) in patients with type 2 diabetes (T2D) remains limited. Therefore, this cohort study aims to explore the association between CVH and AS in patients with T2D.

## SUBJECTS AND METHODS

### Study subjects

The Kailuan Study is a prospective cohort study conducted in the Kailuan community in Tangshan, China. The study was approved by the Ethics Committee of Kailuan General Hospital and conducted in accordance with the Declaration of Helsinki (No. 2006-05). All participants provided written informed consent. For the present study, inclusion criterion was a diagnosis of T2D at baseline assessment. Exclusion criteria were a known history of iAS and missing data. Data of 15,401 participants diagnosed with T2D were extracted from the Kailuan database. Participants diagnosed with iAS at baseline (n = 88) and those with missing data (n = 12,628) were subsequently excluded. Ultimately, 2,685 participants were included in the present study.

#### Assessment of LE8 metrics

In this study, LE8 comprised four health behaviors (diet health, physical activity, nicotine exposure, and sleep health) and four health factors (BMI, blood lipids, blood glucose, and blood pressure). Due to the absence of comprehensive dietary data, salt intake, tea consumption, and fatty food intake were used as surrogate indicators of diet quality, as described in previous studies (^18^,^19^). Data on perceived salt intake, fatty food intake, tea consumption, smoking status, and physical activity were collected using questionnaires. Trained investigators measured anthropometric parameters, including weight and height. A calibrated scale was used to measure body weight (kg) and height (cm) in participants while wearing light clothing and no shoes. All measurements were recorded to the one decimal place. BMI (kg/m^2^) was calculated as weight (kg) divided by height squared (m^2^). Blood samples were collected from the antecubital vein in the morning after an overnight fast and transferred into vacuum tubes containing EDTA. Cholesterol levels were measured en-zymatically. Non-high-density lipoprotein-cholesterol (non-HDL-C) was calculated as follows: non-HDL-C = total cholesterol (TC) – high-density lipoprotein-cholesterol (HDL-C). Fasting blood glucose was assessed using the hexokinase/glucose-6-phosphate dehydro-genase method. Blood pressure was measured on the left arm using a mercury sphygmomanometer with an appropriately sized cuff and recorded to the nearest 2 mmHg. Two measurements of systolic and diastolic blood pressure were obtained at five-minute intervals after participants had rested in a seated position for at least five minutes. The average of the two readings was used for analysis. If the difference between the two measurements exceeded 5 mmHg, an additional reading was conducted, and the average of all three readings was used. The LE8 behavior or factor scores ranged from 0 to 100. The overall LE8 score was calculated as the unweighted average of the individual behavior or factor, yielding a total score ranging from 0 to 100. Component metrics are defined and scored in **Supplementary Table 1.**

**Table 1:** Baseline characteristics

**Variables**	**Total (n = 2,685)**	**Q1(n =671)**	**Q2 (n = 671)**	**Q3 (n = 672)**	**Q4(n =671)**	***P* Value**
Age, years	55.24 ± 10.85	56.60 ± 10.29	55.65 ± 10.70	54.85 ± 10.62	53.88 ± 11.59	<0.001
Male, n (%)	2,118 (78.88)	576 (85.84)	552 (82.27)	544 (80.95)	446 (66.47)	<0.001
BMI, kg/m^2^	26.03 ± 3.43	27.04 ± 3.64	26.19 ± 3.30	25.68 ± 3.18	25.22 ± 3.33	<0.001
SBP, mmHg	135.97 ± 18.96	141.72 ± 17.98	138.89 ± 18.87	133.25 ± 17.43	130.04 ± 19.28	<0.001
DBP, mmHg	85.83 ± 10.87	88.25 ± 10.89	87.47 ± 11.23	85.12 ± 10.16	82.49 ± 10.26	<0.001
FBG, mmol/L	7.85 ± 3.03	8.30 ± 2.72	8.00 ± 2.64	7.69 ± 3.84	7.42 ± 2.67	<0.001
TC, mmol/L	5.18 ± 1.51	5.63 ± 1.28	5.30 ± 2.24	4.98 ± 0.98	4.81 ± 1.05	<0.001
TG, mmol/L	1.58 (1.10-2.54)	1.95 (1.31-3.18)	1.61 (1.12-2.59)	1.49 (1.06-2.32)	1.34 (0.94-2.17)	<0.001
HDL-C, mmol/L	1.53 ± 0.74	1.58 ± 1.24	1.49 ± 0.44	1.52 ± 0.48	1.54 ± 0.49	0.234
LDL-C, mmol/L	2.72 ± 0.90	2.86 ± 0.81	2.76 ± 0.80	2.71 ± 1.17	2.56 ± 0.72	<0.001
Non-HDL-C, mmol/L	3.65 ± 1.56	4.05 ± 1.69	3.81 ± 2.10	3.47 ± 0.97	3.26 ± 1.11	<0.001
hs-CRP, mg/L	1.48 (0.64-3.74)	1.60 (0.80-3.79)	1.50 (0.70-3.50)	1.38 (0.56-3.86)	1.36 (0.52-3.84)	0.355
Current smoker, n (%)	957 (35.64)	363 (54.10)	259 (38.60)	217 (32.29)	118 (17.59)	<0.001
Current drinker, n (%)	1,039 (38.70)	322 (47.99)	267 (39.79)	245 (36.46)	205 (30.55)	<0.001
Income >1,000 yuan/month, n (%)	1,420 (52.89)	427 (63.64)	357 (53.20)	336 (50.00)	300 (44.71)	<0.001
High educational, n (%)	781 (29.09)	183 (27.27)	180 (26.83)	189 (28.13)	229 (34.13)	0.023
Dyslipidemia, n (%)	1,823 (67.90)	552 (82.27)	483 (71.98)	427 (63.54)	361 (53.80)	<0.001
Hypertension, n (%)	1,608 (59.89)	505 (75.26)	450 (67.06)	354 (52.68)	299 (44.56)	<0.001
Hypolipidemic drugs, n (%)	546 (20.34)	167 (24.89)	151 (22.50)	117 (17.41)	111 (16.54)	0.001
Hypotensive drugs, n (%)	935 (34.82)	328 (48.88)	258 (38.45)	180 (26.79)	169 (25.19)	<0.001
Oral antidiabetic drugs, n (%)	1,287 (47.93)	349 (52.01)	317 (47.24)	308 (45.83)	313 (46.65)	0.182
Insulin, n (%)	420 (15.64)	120 (17.88)	84 (12.52)	102 (15.18)	114 (16.99)	0.013

BMI: body mass index; SBP: systolic blood pressure; DBP: diastolic blood pressure; FBG: fasting blood glucose; TC: total cholesterol; TG: triglycerides; HDL-C: high-density lipoprotein cholesterol; LDL-C: low-density lipoprotein cholesterol; Non-HDL-C: non-high density lipoprotein cholesterol; hs-CRP: high sensitivity C-reactive protein.

The cumulative exposure to LE8 (cum-LE8) score was calculated as (LE82006+LE82008)/2xtime2006-2008+(LE 82008+LE82010)/2xtime2008-2010' in which LE82006' LE82008' and LE82010 represented LE8 scores obtained during the first (2006-2007), second (2008-2009), and third (2010-2011) health examinations, respectively. Time2006-2008 and time2008-2010 represented the time interval, in years, between two consecutive visits. The mean time2006-2008 and time2008-2010 were 2.10 and 1.92 years, respectively. Participants were stratified by the quartiles of cum-LE8 score: quartile 1 (Q1) (≤ 102.08), quartile 2 (Q2) (102.09-117.98), quartile 3 (Q3) (117.99-135.47), and quartile 4 (Q4) (> 135.47).

#### Measurement of baPWV and iAS diagnosis

Brachial-ankle pulse wave velocity (baPWV) served as an index for evaluating AS. BaPWV was measured using a BP-203 RPE III device (Omron Health Medical [China] Co., LTD). Measurements were performed twice at five-minute intervals, and the second measurement was used as the final value. In this study, BaPWV ≥ A 1800 cm/s was defined as iAS (^20^). Follow-up continued until the first occurrence of iAS, all-cause mortality, or the end of follow-up on December 31, 2021, whichever occurred first.

#### Statistical analysis

All analyses were performed with SAS v.9.4 (SAS Institute, Inc, Cary, NC, USA). The Kaplan-Meier method was used to calculate the cumulative incidence of iAS, and the log-rank test was used to assess differences between groups. Cox proportional hazards regression model was used to analyze the association between cum-LE8 and the risk of iAS. Sensitivity analyses were performed by removing each component of LE8. In addition, restricted cubic spline (RCS) analysis was conducted to explore the dose-response relationship between the cum-LE8 and the risk of iAS. A two-sided *P* < 0.05 was considered statistically significant.

### RESULTS

#### Baseline characteristics

The mean age of the study participants was 55.24 years, of which 2,118 participants (78.88%) were male, and the mean cum-LE8 score was 119.94. With increment in the cum-LE8 score' the age' the proportion of men, the levels of BMI, and blood pressure significantly decreased **(Table 1).** Baseline characteristics stratified by sex are presented in **Supplementary Table 2** and **Table 3,** respectively.

**Table 2:** Hazard ratios and 95% confidence intervals for iAS

	**HR (95% CI)**		
	**Model 1**	**Model 2**	**Model 3**
Q1	1	1	1
Q2	0.95 (0.82-1.10)	0.96 (0.83-1.11)	0.69 (0.59-0.80)
Q3	0.78 (0.67-0.91)	0.82 (0.70-0.95)	0.41 (0.35-0.49)
Q4	0.71 (0.61-0.82)	0.76 (0.65-0.89)	0.15 (0.12-0.18)
Per SD increase	0.90 (0.85-0.95)	0.92 (0.87-0.97)	0.48 (0.45-0.51)
*P* for trend	<0.001	<0.001	<0.001

Model 1 was unadjusted. Model 2 was adjusted for age and sex. Model 3 was adjusted for age, sex, duration of diabetes, habit of drinking alcohol, high sensitivity C-reactive protein, income level and education level. iAS: increased arterial stiffness; HR: hazard ratio; CI: confidence interval; SD: standard deviation.

**Table 3:** Sensitivity analysis

**Removed component**	**HR (95% CI)**				**Per SD increase**	***P* for trend**
	**Q1**	**Q2**	**Q3**	**Q4**		
Diet health	1	0.67 (0.58-0.78)	0.43 (0.37-0.51)	0.16 (0.13-0.19)	0.49 (0.46-0.52)	<0.001
Physical activity	1	0.61 (0.53-0.71)	0.38 (0.32-0.44)	0.15 (0.13-0.18)	0.47 (0.45-0.50)	<0.001
Sleep health	1	0.67 (0.58-0.78)	0.48 (0.41-0.56)	0.17 (0.14-0.21)	0.51 (0.49-0.55)	<0.001
Nicotine exposure	1	0.61 (0.53-0.71)	0.41 (0.35-0.47)	0.16 (0.14-0.20)	0.51 (0.49-0.55)	<0.001
Body mass index	1	0.72 (0.62-0.84)	0.47 (0.40-0.55)	0.17 (0.14-0.20)	0.51 (0.48-0.54)	<0.001
Blood lipids	1	0.75 (0.64-0.87)	0.48 (0.41-0.56)	0.18 (0.15-0.21)	0.50 (0.47-0.53)	<0.001
Blood glucose	1	0.61 (0.52-0.71)	0.46 (0.39-0.54)	0.17 (0.14-0.20)	0.51 (0.48-0.54)	<0.001
Blood pressure	1	0.69 (0.59-0.81)	0.51 (0.43-0.60)	0.17 (0.14-0.20)	0.50 (0.48-0.54)	<0.001

Model was adjusted for age, sex, duration of diabetes, habit of drinking alcohol, high sensitivity C-reactive protein, income level and education level. iAS: increased arterial stiffness; HR: hazard ratio; CI: confidence interval; SD: standard deviation.

#### Incidence rate and cumulative incidence of iAS

Over a median follow-up of 9.76 years, we identified 1,375 cases of iAS. Specifically, 381, 370, 326, and 298 cases occurred in Q1, Q2, Q3, and Q4, respectively. The incidence rate of iAS in Q1, Q2, Q3, and Q4 were 71.02 (64.24-78.52), 67.68 (61.13-74.94), 56.05 (50.28-62.47), and 50.47 (45.05-56.53) per 1,000 person-years, respectively. The Kaplan-Meier curve showed that the cumulative incidence of iAS was highest in Q1 and gradually decreased from Q1 to Q4 (log-rank *P* < 0.01) **(Figure 1).** Cumulative incidence for men and women is shown in **Supplementary Figure 1.**

**Figure 1:**
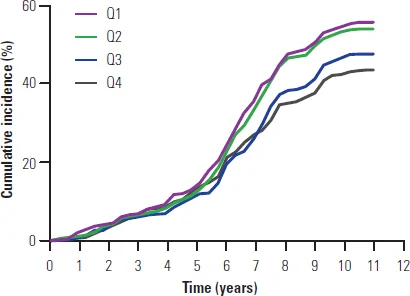
Kaplan-Meier curves of iAS according to cum-LE8 scores quartiles.

iAS: increased arterial stiffness.

#### Hazard ratios and 95% confidence intervals for iAS

In the full adjusted model, compared with Q1, hazard ratios (HRs) and 95% confidence intervals (CIs) for the Q2, Q3, and Q4 groups were 0.69 (0.59-0.80), 0.41 (0.35-0.49), and 0.15 (0.12-0.18), respectively. Additionally' each standard deviation increase in cum-LE8 score was associated with an HR (95% CI) for iAS of 0.48 (0.45-0.51) **(Table 2).** Hazard ratios and 95% confidence intervals for iAS stratified by sex are presented in **Supplementary Table 4** and **Table 5,** respectively. Sensitivity analyses yielded similar results **(Table 3),** results stratified by sex are presented in **Supplementary Tables 6** and **Table 7,** respectively. Furthermore, multivariate-adjusted RCS analysis showed a linear association between cum-LE8 score and iAS *(P* for overall < 0.001, *P* for non-linearity = 0.051) **(Figure 2).** RCS analysis for men and women is shown in **Supplementary Figure 2.**

**Figure 2:**
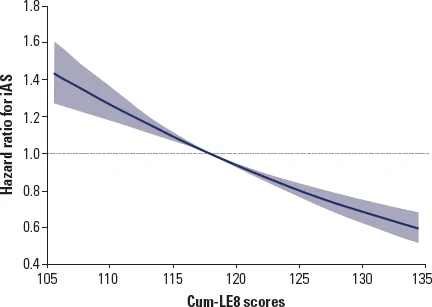
Restricted cubic spline analysis for the risk of iAS according to cum-LE8 scores quartiles.

Model was adjusted for age, sex, duration of diabetes, habit of drinking alcohol, high sensitivity C-reactive protein, income level and education level. Five knots were placed at 5th, 25th, 50th, 75th, and 95th percentiles of cum-LE8 scores distribution.

iAS: increased arterial stiffness; HR: hazard ratio.

### DISCUSSION

Accumulating evidence suggests a strong correlation between AS and the progression of diabetes-related complications. Moreover, AS may also be elevated in pre-diabetic groups with impaired glucose tolerance (^21^). In this study' we explored the association between adherence to CVH and AS. To our knowledge, this is the first study to investigate the association between LE8 and new-onset iAS in patients with T2D. Our results indicate that increases in the cum-LE8 score were associated with a gradual reduction in the cumulative incidence of iAS among patients with T2D. Improved long-term CVH status was associated with a lower risk of iAS.

Because far more men than women were employees of the Kailuan Group, one of China's major coal production and consumption enterprises' approximately 80% of participants with type 2 diabetes in this cohort were male. However, the prevalence of diabetes in China is comparable between women and men, with a prevalence of 12.1% among men and 11.0% among women (^22^). Therefore, these findings may not apply to other populations' particularly women and non-Chinese ethnic groups. Extensive research over the decades has established gender differences in the onset of CVDs, particularly regarding the timing and degree of arterial stiffening. Furthermore, mechanistic data identifying factors contributing to iAS in conditions more prevalent among women, such as preeclampsia' obesity' and autoimmune diseases' remain limited. Considering the significance of arterial stiffening in the progression of CVDs and the evidence indicating gender differences in CVDs, further studies are warranted (^23^).

Consistent with our results, previous research has shown that moderate and low CVH levels were linked to a higher incidence of elevated baPWV compared to high CVH, indicating an inverse relationship between LE8 scores and PWV (^24^,^25^,^26^,^27^). Because higher scores on both LE8 and LS7 scales reflect greater adherence to AHA guidelines, analyses based on LS7 scores produced comparable outcomes (28-33). It should be noted that baPWV is a convenient and increasingly used method for assessing AS. However, carotid-femoral pulse wave velocity (cfPWV) remains the gold standard measurement of AS (^34^). Indeed, several previous studies on this topic evaluated cfPWV rather than baPWV (^27^,^28^). A prior study reported that average baPWV values were approximately 20% higher than cfPWV values. Nonetheless, both cfPWV and baPWV are recognized indices of AS and have demonstrated similar associations with CVDs risk factors and clinical outcomes (^35^).

Our study has some limitations. First, we calculated the progression of baPWV based on the first and last baPWV measurements obtained for each participant. Because the time interval between these measurements varied among participants' this may have influenced the results. Second, to assess CVH at the population level, the Dietary Approaches to Stop Hypertension (DASH) diet score is recommended within LE8 to evaluate dietary habits in both adult and pediatric populations (^16^,^36^). This score comprises eight components: high intake of fruits, vegetables, nuts, and legumes, whole grains, and low-fat dairy products, alongside a reduced intake of sodium, red and processed meats, and sugar-sweetened beverages. However, except for sodium intake, information on other components were unavailable in our study. Therefore, intake of high-fat foods and tea consumption obtained from questionnaires were used as surrogate indicators of diet quality. Nevertheless, high-fat foods were neither classified nor quantified. Additionally, the assessment of physical activity and nicotine exposure was not fully consistent with AHA criteria, which may have introduced measurement bias. Specifically, AHA criteria classify physical activity according to the frequency of moderate- or vigorous-intensity activity (^16^); however, information regarding exercise intensity was unavailable in our study. Regarding nicotine exposure, AHA criteria classify former smokers according to smoking cessation duration, but do not quantify cigarette consumption among current smokers (^16^). In contrast, our study quantified cigarette consumption among current smokers; however, only former smokers who had quit for more than one year were recorded. Third, because the cohort was not nationally representative, the findings may not apply to other populations.

In conclusion, increased compliance with the AHA's LE8 is linked to a reduced risk of iAS. Furthermore, achieving a higher long-term CVH status is associated with a reduced risk of iAS. LE8 may serve as a valuable indicator for assessing the risk of iAS in patients with T2D.

## Figures and Tables

**Supplementary Table 1:** Definition and quantitative scoring of cardiovascular health (Kailuan version of Life's Essential 8)

**Components**	**Method of measurement**	**Quantile**	**Points**
Diet health	Self-reported intake of salt per day	< 6 g	100
		6-12 g	50
		> 12 g	0
	Self-reported intake of fatty foods per week	< 1 time	100
		1-3 times	50
		> 3 times	0
		≥ 4 times/week	100
	Self-reported tea consumption	1-3 times/week	75
		1-3 times/month	50
		< 1 time/month	25
		Never	0
Physical activity	Self-reported times of physical activity per week	> 60 min	100
		20-60 minutes	50
		< 20 minutes	0
Nicotine exposure	Self-reported use of cigarettes	Never smokers	100
		Former smokers with quit years >1	50
		Current smoker (< 1 cigarette/day)	25
		Current smoker (> 1 cigarette /day)	0
Sleep health	Self-reported average hours of sleep per night	7~< 9 hours	100
		9~< 10 hours	90
		6~< 7 hours	70
		5~< 6 or ≥ 10 hours	40
		4~< 5 hours	20
		< 4 hours	0
BMI	Body weight and height squared with calculation of BMI	< 23.0 kg/m^2^	100
		23.0-24.9 kg/m^2^	75
		25.0-29.9 kg/m^2^	50
		30.0-34.9 kg/m^2^	25
		≥ 35.0 kg/m^2^	0
Blood lipids	Plasma total and HDL cholesterol with calculation of non-HDL cholesterol	< 3.36 mmol/L	100
		3.36-4.13 mmol/L	60
		4.14-4.90 mmol/L	40
		4.91-5.68 mmol/L	20
		≥ 5.69 mmol/L	0
			Subtract 20 points if drug-treated
Blood glucose	Measured FBG	No diabetes and FBG < 5.6 mmol/L	100
		No diabetes and FBG 5.6-6.9 mmol/L	60
		Diabetes and FBG < 8.6 mmol/L	40
		Diabetes and FBG 8.6-10.1 mmol/L	30
		Diabetes and FBG 10.2-11.6 mmol/L	20
		Diabetes and FBG 11.7-13.2 mmol/L	10
		Diabetes and FBG ≥ 13.3 mmol/L	0
Blood pressure	Measured systolic and diastolic blood pressures	SBP < 120 and DBP < 80 mmHg	100
		SBP 120-129 and DBP < 80 mmHg	75
		SBP 130-139 or DBP 80-89 mmHg	50
		SBP 140-159 or DBP 90-99 mmHg	25
		SBP ≥ 160 or DBP ≥ 100 mmHg	0
			Subtract 20 points if drug-treated

Abbreviations: BMI: body mass index; Non-HDL-C: non-high density lipoprotein cholesterol; FBG: fasting blood glucose; SBP: systolic blood pressure; DBP: diastolic blood pressure.

**Supplementary Table 2:** Baseline characteristics (men)

**Variables**	**Total (n = 2,118)**	**Q1 (n = 576)**	**Q2 (n = 552)**	**Q3 (n = 544)**	**Q4(n = 446)**	***P* Value**
Age, years	54.80 ± 10.80	55.51 ± 10.09	54.86 ± 10.81	54.29 ± 10.66	54.42 ± 11.78	0.146
BMI, kg/m^2^	26.07 ± 3.42	27.03 ± 3.61	26.16 ± 3.26	25.68 ± 3.21	25.21 ± 3.33	<0.001
SBP, mmHg	136.11 ± 17.95	141.63 ± 17.76	138.10 ± 17.86	133.11 ± 17.33	130.18 ± 16.54	<0.001
DBP, mmHg	86.69 ± 10.72	89.16 ± 10.88	88.05 ± 10.87	85.51 ± 10.11	83.25 ± 9.93	<0.001
FBG, mmol/L	7.93 ± 3.12	8.32 ± 2.68	8.00 ± 2.61	7.80 ± 4.12	7.50 ± 2.72	0.001
TC, mmol/L	5.14 ± 1.57	5.55 ± 1.26	5.22 ± 2.38	4.93 ± 0.97	4.77 ± 1.05	<0.001
TG, mmol/L	1.59 (1.10-2.63)	1.98 (1.32-3.29)	1.59 (1.10-2.61)	1.50 (1.04-2.35)	1.35 (0.94-2.20)	<0.001
HDL-C, mmol/L	1.51 ± 0.81	1.57 ± 1.33	1.47 ± 0.45	1.49 ± 0.48	1.53 ± 0.52	0.297
LDL-C, mmol/L	2.69 ± 0.91	2.81 ± 0.79	2.69 ± 0.75	2.68 ± 1.25	2.54 ± 0.67	<0.001
Non-HDL-C, mmol/L	3.63 ± 1.64	3.99 ± 1.74	3.75 ± 2.22	3.44 ± 0.96	3.24 ± 1.14	<0.001
hs-CRP, mg/L	1.50 (0.60-3.80)	1.65 (0.80-3.93)	1.41 (0.60-3.36)	1.40 (0.50-4.12)	1.40 (0.50-4.10)	0.333
Current smoker, n (%)	930 (43.91)	360 (62.50)	249 (45.11)	212 (38.97)	109 (24.44)	<0.001
Current drinker, n (%)	1,001 (47.26)	319 (55.38)	260 (47.10)	239 (43.93)	183 (41.03)	<0.001
Income >1,000 yuan/month, n (%)	1,084 (51.18)	340 (59.03)	280 (50.72)	264 (48.53)	200 (44.84)	<0.001
High educational, n (%)	584(27.57)	145 (25.17)	146 (26.45)	146 (26.84)	147 (32.96)	0.023
Dyslipidemia, n (%)	1,439(67.94)	470 (81.60)	387 (70.11)	347 (63.79)	235 (52.69)	<0.001
Hypertension, n (%)	1,299 (61.33)	429 (74.48)	372 (67.39)	294 (54.04)	204 (45.74)	<0.001
Hypolipidemic drugs, n (%)	397 (18.74)	134 (23.26)	108 (19.57)	89 (16.36)	66 (14.80)	0.006
Hypotensive drugs, n (%)	695 (32.81)	262 (45.49)	188 (34.06)	141 (25.92)	104 (23.32)	<0.001
Oral antidiabetic drugs, n (%)	947 (44.71)	275 (47.74)	249 (45.11)	232 (42.65)	191 (42.83)	0.735
Insulin, n (%)	293 (13.83)	91 (15.80)	59 (10.69)	75 (13.79)	68 (15.25)	0.035

Abbreviations: BMI: body mass index; SBP: systolic blood pressure; DBP: diastolic blood pressure; FBG: fasting blood glucose; TC: total cholesterol; TG: triglycerides; HDL-C: high-density lipoprotein cholesterol; LDL-C: low-density lipoprotein cholesterol; Non-HDL-C: non-high density lipoprotein cholesterol; hs-CRP: high sensitivity C-reactive protein.

**Supplementary Table 3:** Baseline characteristics (women)

**Variables**	**Total (n = 567)**	**Q1 (n = 95)**	**Q2(n = 119)**	**Q3(n = 128)**	**Q4 (n = 225)**	***P* Value**
Age, years	56.90 ± 10.90	63.22 ± 8.99	59.31 ± 9.39	57.21 ± 10.19	52.80 ± 11.14	<0.001
BMI, kg/m^2^	25.87 ± 3.45	27.13 ± 3.79	26.29 ± 3.49	25.67 ± 3.06	25.24 ± 3.34	<0.001
SBP, mmHg	135.47 ± 22.33	142.31 ± 19.35	142.57 ± 22.72	133.83 ± 17.92	129.77 ± 23.84	<0.001
DBP, mmHg	82.63 ± 10.85	82.76 ± 9.22	84.74 ± 12.44	83.45 ± 10.24	80.99 ± 10.74	0.033
FBG, mmol/L	7.56 ± 2.63	8.22 ± 2.94	7.98 ± 2.79	7.21 ± 2.25	7.27 ± 2.55	0.007
TC, mmol/L	5.33 ± 1.25	6.14 ± 1.33	5.65 ± 1.38	5.20 ± 0.98	4.89 ± 1.05	<0.001
TG, mmol/L	1.54 (1.10-2.28)	1.91 (1.31-3.01)	1.64 (1.21-2.44)	1.46 (1.09-2.21)	1.31 (0.94-2.14)	<0.001
HDL-C, mmol/L	1.61 ± 0.41	1.67 ± 0.40	1.59 ± 0.37	1.63 ± 0.44	1.58 ± 0.40	0.404
LDL-C, mmol/L	2.85 ± 0.86	3.13 ± 0.91	3.08 ± 0.94	2.84 ± 0.73	2.61 ± 0.79	<0.001
Non-HDL-C, mmol/L	3.72 ± 1.23	4.47 ± 1.30	4.06 ± 1.37	3.56 ± 0.98	3.31 ± 1.05	<0.001
hs-CRP, mg/L	1.40 (0.70-3.34)	1.00 (0.62-2.90)	2.15 (1.00-4.00)	1.17 (0.60-2.84)	1.30 (0.64-3.12)	0.031
Current smoker, n (%)	27 (4.76)	3 (3.16)	10 (8.40)	5 (3.91)	9 (4.00)	0.340
Current drinker, n (%)	38 (6.70)	3 (3.16)	7 (5.88)	6 (4.69)	22 (9.78)	0.179
Income >1,000 yuan/month, n (%)	336 (59.26)	87 (91.58)	77 (64.71)	72 (56.25)	100 (44.44)	<0.001
High educational, n (%)	197 (34.74)	38 (40.00)	34 (28.57)	43 (33.59)	82 (36.44)	0.475
Dyslipidemia, n (%)	384 (67.72)	82 (86.32)	96 (80.67)	80 (62.50)	126 (56.00)	<0.001
Hypertension, n (%)	309 (54.50)	76 (80.00)	78 (65.55)	60 (46.88)	95 (42.22)	<0.001
Hypolipidemic drugs, n (%)	340 (59.96)	74 (77.89)	68 (57.14)	76 (59.38)	122 (54.22)	0.003
Hypotensive drugs, n (%)	240 (42.33)	66 (69.47)	70 (58.82)	39 (30.47)	65 (28.89)	<0.001
Oral antidiabetic drugs, n (%)	340 (59.96)	74 (77.89)	68 (57.14)	76 (59.38)	122 (54.22)	0.003
Insulin, n (%)	127 (22.40)	29 (30.53)	25 (21.01)	27(21.09)	46 (20.44)	0.359

Abbreviations: BMI: body mass index; SBP: systolic blood pressure; DBP: diastolic blood pressure; FBG: fasting blood glucose; TC: total cholesterol; TG: triglycerides; HDL-C: high-density lipoprotein cholesterol; LDL-C: low-density lipoprotein cholesterol; Non-HDL-C: non-high density lipoprotein cholesterol; hs-CRP: high sensitivity C-reactive protein.

**Supplementary Table 4:** Hazard ratios and 95% confidence intervals for iAS (men)

	**HR (95% CI)**		
	**Model 1**	**Model 2**	**Model 3**
Q1	1	1	1
Q2	1.01 (0.86-1.18)	1.01 (0.86-1.19)	0.73 (0.62-0.86)
Q3	0.83 (0.70-0.98)	0.85 (0.72-1.00)	0.47 (0.39-0.55)
Q4	0.78 (0.65-0.94)	0.81 (0.68-0.98)	0.19 (0.15-0.23)
Per SD increase	0.91 (0.85-0.98)	0.93 (0.86-0.99)	0.54 (0.51-0.58)
*P* for trend	0.001	0.001	<0.001

Model 1 was unadjusted. Model 2 was adjusted for age and sex. Model 3 was adjusted for age, sex, duration of diabetes, habit of drinking alcohol, high sensitivity C-reactive protein, income level and education level. Abbreviations: HR: hazard ratio; CI: confidence interval; SD: standard deviation.

**Supplementary Table 5:** Hazard ratios and 95% confidence intervals for iAS (women)

	**HR (95% CI)**		
	**Model 1**	**Model 2**	**Model 3**
Q1	1	1	1
Q2	0.66 (0.47-0.91)	0.77 (0.56-1.08)	0.50 (0.34-0.73)
Q3	0.53 (0.38-0.73)	0.68(0.48-0.95)	0.19 (0.13-0.28)
Q4	0.40 (0.30-0.54)	0.60 (0.43-0.82)	0.06 (0.04-0.09)
Per SD increase	0.80 (0.72-0.89)	0.91 (0.82-1.01)	0.32 (0.27-0.38)
*P* for trend	<0.001	0.002	<0.001

Model 1 was unadjusted. Model 2 was adjusted for age and sex. Model 3 was adjusted for age, sex, duration of diabetes, habit of drinking alcohol, high sensitivity C-reactive protein, income level and education level. Abbreviations: HR: hazard ratio; CI: confidence interval; SD: standard deviation.

**Supplementary Table 6:** Sensitivity analysis (men)

**Removed component**	**HR (95% CI)**				**Per SD increase**	***P* for trend**
	**Q1**	**Q2**	**Q3**	**Q4**		
Diet health	1	0.69 (0.59-0.82)	0.47 (0.40-0.56)	0.19 (0.16-0.24)	0.55 (0.52-0.58)	<0.001
Physical activity	1	0.66 (0.56-0.78)	0.45 (0.38-0.53)	0.19 (0.16-0.23)	0.54 (0.51-0.58)	<0.001
Sleep health	1	0.66 (0.56-0.78)	0.51 (0.43-0.61)	0.21 (0.18-0.26)	0.58 (0.54-0.61)	<0.001
Nicotine exposure	1	0.73 (0.61-0.86)	0.44 (0.37-0.53)	0.19 (0.15-0.23)	0.56 (0.53-0.60)	<0.001
Body mass index	1	0.69 (0.59-0.82)	0.53 (0.45-0.63)	0.22 (0.18-0.26)	0.57 (0.54-0.61)	<0.001
Blood lipids	1	0.78(0.66-0.91)	0.47 (0.40-0.56)	0.22 (0.18-0.27)	0.56 (0.52-0.60)	<0.001
Blood glucose	1	0.59 (0.50-0.69)	0.50 (0.42-0.59)	0.21 (0.17-0.25)	0.57 (0.53-0.60)	<0.001
Blood pressure	1	0.68(0.57-0.81)	0.55 (0.46-0.66)	0.22 (0.18-0.26)	0.57 (0.53-0.60)	<0.001

Model was adjusted for age, sex, duration of diabetes, habit of drinking alcohol, high sensitivity C-reactive protein, income level and education level. Abbreviations: HR: hazard ratio; CI: confidence interval; SD: standard deviation.

**Supplementary Table 7:** Sensitivity analysis (women)

**Removed component**	**HR (95% CI)**				**Per SD increase**	***P* for trend**
	**Q1**	**Q2**	**Q3**	**Q4**		
Diet health	1	0.55 (0.37-0.80)	0.23 (0.16-0.33)	0.07 (0.05-0.11)	0.33 (0.28-0.38)	<0.001
Physical activity	1	0.38 (0.26-0.55)	0.15 (0.10-0.22)	0.06 (0.04-0.08)	0.29 (0.25-0.35)	<0.001
Sleep health	1	0.55 (0.37-0.81)	0.28 (0.19-0.41)	0.07 (0.05-0.11)	0.35 (0.30-0.41)	<0.001
Nicotine exposure	1	0.31 (0.22-0.44)	0.24 (0.16-0.34)	0.07 (0.05-0.11)	0.35 (0.30-0.41)	<0.001
Body mass index	1	0.65 (0.45-0.95)	0.21 (0.14-0.30)	0.06 (0.04-0.09)	0.34 (0.29-0.40)	<0.001
Blood lipids	1	0.45 (0.30-0.67)	0.33 (0.22-0.49)	0.08 (0.05-0.12)	0.37 (0.32-0.43)	<0.001
Blood glucose	1	0.64 (0.44-0.93)	0.24 (0.17-0.36)	0.08 (0.05-0.11)	0.35 (0.30-0.41)	<0.001
Blood pressure	1	0.78 (0.54-1.12)	0.29 (0.20-0.41)	0.07 (0.05-0.10)	0.34 (0.29-0.40)	<0.001

Model was adjusted for age, sex, duration of diabetes, habit of drinking alcohol, high sensitivity C-reactive protein, income level and education level. Abbreviations: HR: hazard ratio; CI: confidence interval; SD: standard deviation.

**Supplementary Figure 1:**
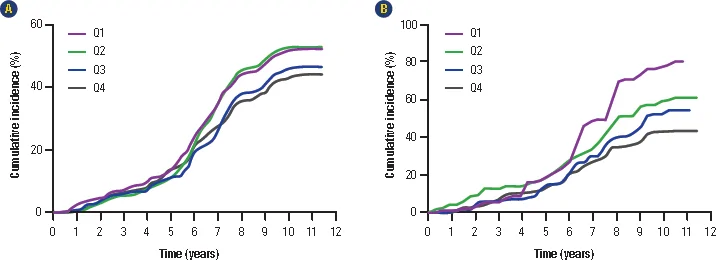
Kaplan-Meier curves of iAS according to cum-LE8 scores quartiles **A** (men), **B** (women).

Abbreviation: iAS: increased arterial stiffness.

**Supplementary Figure 2:**
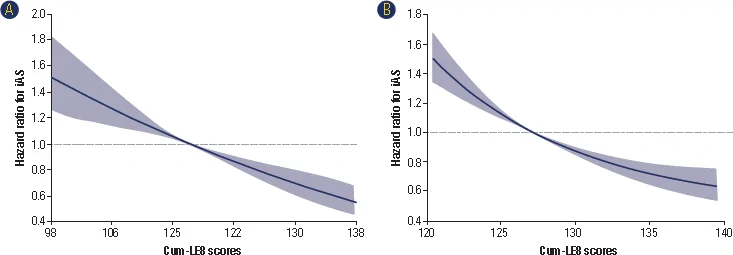
Restricted cubic spline analysis for the risk of iAS according to quartiles of cum-LE8 scores **A** (men), **B** (women).

Abbreviation: iAS: increased arterial stiffness; HR: hazard ratio.

## SUPPLEMENTARY MATERIALS

Model was adjusted for age, sex, duration of diabetes, habit of drinking alcohol, high sensitivity C-reactive protein, income level and education level. Five knots were placed at 5th, 25th, 50th, 75th, and 95th percentiles of cum-LE8 scores distribution. *P* for overall < 0.001, *P* for non-linearity = 0.098 **(A),** *P* for overall < 0.001, *P* for non-linearity = 0.003 **(B).**
